# Supplementary material for: Antibiotic resistance genes in the gut microbiota of mothers and linked neonates with or without sepsis from low- and middle-income countries
Source: Nat Microbiol. 2022 Aug 4;7(9):1337–47. doi: 10.1038/s41564-022-01184-y (PMC9417982; doi:10.1038/s41564-022-01184-y)
Supplement: Supplementary file 1 — Supplementary Methods and Tables 1–5. [file 41564_2022_1184_MOESM1_ESM.pdf]

nature portfolio

https://doi.org/10.1038/s41564-022-01184-y

Supplementary information

Antibiotic resistance genes in the gut microbiota of mothers and linked neonates with or without sepsis from low- and middle-income countries

In the format provided by the authors and unedited

Supplementary Material Methods: Exploratory multivariable analyses performed

Association between WASH (water, sanitation and hygiene) related variables and maternal carriage of *bla*$_{CTX-M-15}$ or *bla*$_{NDM}$ or *bla*$_{OXA-48}$-like genes

Logistic regression models were fitted to each ARG (outcome), including the following explanatory variables:

- Residence water supply
- Drinking water
- Residence access to wastewater network
- Frequency of solid waste collection
- Access to soap
- Mother handwashing frequency
- Mother showering/bathing frequency → excluded due to high number of missing responses
- Type of residence*
- Type of toilet in residence
- Number of people living in residence*
- Number of bedrooms in residence*
- Household income equal or higher than country average
- Mother education status

*Models were fitted including and excluding these variables.

Association between the mother's handwashing frequency and maternal carriage of *bla*$_{CTX-M-15}$ or *bla*$_{NDM}$ or *bla*$_{OXA-48}$-like genes

Logistic regression models fitted to each ARG (outcome), included handwashing frequency as the explanatory variable and controlling for the following variables:

- Mother education status
- Household income equal or higher than country average
- Residence water supply
- Access to soap
- Frequency of solid waste collection
- Residence access to wastewater network
- Type of residence*

*Models were fitted including and excluding these variables.

Association between maternal infection in the three-months prior to enrolment in the study and maternal carriage of *bla*$_{CTX\text{-}M\text{-}15}$ or *bla*$_{NDM}$ or *bla*$_{OXA\text{-}48}$-like genes

Logistic regression models fitted to each ARG (outcome), included prior maternal infection as the explanatory variable and controlling for the following variables:

- Age of mother
- Mother use of antibiotics in the three months prior to enrolment*
- Mother visited hospital in the 12 months prior to enrolment
- Mother attended private healthcare in the three months prior to enrolment
- Mother visited traditional healer in the three months prior to enrolment
- Household income equal or higher than country average
- Mother education status
- Type of toilet in residence
- Mother handwashing frequency
- Residence access to wastewater network
- Frequency of solid waste collection
- Residence water supply
- Mother underlying diseases: immunocompromised
- Mother underlying diseases: diabetes

*Models were fitted with and without this variable due to the possibility of being a confounder and/or a mediator variable.

Association between maternal use of antibiotics in the three-months prior to enrolment in the study and maternal carriage of *bla*$_{CTX\text{-}M\text{-}15}$ or *bla*$_{NDM}$ or *bla*$_{OXA\text{-}48}$-like genes

Logistic regression model fitted to each ARG (outcome), included prior maternal use of antibiotics as the explanatory variable and controlling for the following variables:

- Maternal infection in the three months prior to enrolment
- Mother underlying diseases: immunocompromised
- Mother underlying diseases: diabetes
- Mother visited hospital in the 12 months prior to enrolment
- Mother attended private healthcare in the three months prior to enrolment
- Mother visited traditional healer in the three months prior to enrolment
- Household income equal or higher than country average
- Mother education status

Association between birth healthcare environment related variables and carriage of *bla*$_{CTX-M-15}$ or *bla*$_{NDM}$ or *bla*$_{OXA-48}$-like genes among neonates of the birth cohort

Logistic regression models were fitted to each ARG (outcome), including the following explanatory variables:

- Number of beds on ward
- Bathroom on ward
- Mother location on ward → excluded due to high number of missing responses
- Delivery type
- Timing of birth*
- Breech
- Perinatal asphyxia
- PPROM*

*Models were fitted either with “Timing of birth” or “PPROM” due to their collinear nature.

Association between age of infant at sample collection and carriage of *bla*$_{CTX-M-15}$ or *bla*$_{NDM}$ or *bla*$_{OXA-48}$-like genes among neonates of the birth cohort

Logistic regression model fitted to each ARG (outcome), included a categorised age at sample collection as the explanatory variable (0-72 hours; ≥72 hours - 60 days) and controlling for the following variables:

- Biological sepsis diagnosis
- Mother use of antibiotics in the three months prior to enrolment
- Residence water supply
- Drinking water
- Residence access to wastewater network
- Frequency of solid waste collection
- Access to soap
- Mother handwashing frequency
- Mother showering/bathing frequency → excluded due to high number of missing responses
- Type of residence*
- Type of toilet in residence
- Number of people living in residence*
- Number of bedrooms in residence*
- Household income equal or higher than country average

- Mother education status
- Delivery type
- Timing of birth**
- Breech
- Perinatal asphyxia
- PPROM**

* Models were fitted including and excluding these variables.

** Models were fitted either with “Timing of birth” or “PPROM” due to their collinear nature.

Supplementary Material Table 1 – World bank data for contextual analysis.

World bank data on mortality, poverty and sanitation in the countries included in BARNARDS (https://data.worldbank.org).

World data indicator	Bangladesh	Ethiopia	India	Nigeria	Pakistan	Rwanda	South Africa
**Pregnant women receiving pre-natal care (% population)[+]**	64	62	79	67	86	98	94
**Mortality rate under-5 (per 1,000 live births, 2018 data)**	30	55	37	120	69	35	34
**Number of neonatal deaths (2018 data)**	50,244	99,275	549,227	267,227	251,484	6,215	12,717
**Mortality rate attributed to unsafe water/sanitation and lack of hygiene (per 100,000 population, 2018 data)**	11.9	43.7	18.6	68.6	19.6	19.3	13.7
**International poverty* (less than $1.90 per day) millions of population, %**	23.3M, 14.8%	31.9M, 30.8%	268.7, 21.2%	84.7M, 53.5%	8M, 3.99%	6.6M, 55.5%	10.5M, 18.9%
**Poverty at the national poverty line* Millions of population, %**	38.4M, 24.3%	23.7M, 23.5%	273.8, 21.9%	71M, 46%	48.5M, 24.3%	4.5M, 38.2%	30.3M, 55.5%
**GINI index***	32.4	35	37.8	43	33.5	43.7	63
**People using at least basic sanitation services (% population, 2017 data)**	48	7	60	39	60	67	76
**People using at least basic sanitation services (% urban population, 2017 data)**	51	20	72	48	77	52	76
**People using at least basic sanitation services (% rural population, 2017 data)**	47	4	53	31	50	70	75
**People practicing open defecation (% population)**	0	22	26	20	10	2	1
**People using at least basic drinking water (% population, 2017 data)**	97	41	93	71	91	58	93
**People using at least basic drinking water (% urban population, 2017 data)**	97	80	96	87	94	82	99
**People using at least basic drinking water (% rural population, 2017 data)**	97	31	91	56	90	53	81
**People using safely managed drinking water (% population, 2017 data)**	55	11	No data	20	35	No data	No data
**People with basic handwashing facilities including soap and water (% population, 2017 data)**	34.81	7.96	59.55	41.95	59.61	4.62	43.99
**People with basic handwashing facilities including soap and water (% urban population, 2017 data)**	51.09	23.55	79.76	52.85	83.25	13.42	52.56
**People with basic handwashing facilities including soap and water (% rural population, 2017 data)**	25.71	4.04	49.32	31.26	46.05	2.8	27.48
**Diarrhoea treatment (% of children under 5 who received ORS packets)****	77	29.5	50.6	40	37.4	27.5	51.4

[+]Date of information: Bangladesh 2014, Ethiopia, 2016, India 2016, Nigeria 2018, Pakistan 2018, Rwanda 2017, South Africa 2016

*Date of information: Bangladesh 2016, Ethiopia 2015, India 2011, Nigeria 2009, Rwanda 2016, Pakistan 2015, South Africa 2014.

**Date of information: Bangladesh 2014, Ethiopia 2016, India 2016, Nigeria 2018, Pakistan 2018, Rwanda 2015, South Africa 2016.

Average household income reported by sites	US dollars
BC (Bangladesh)	$150
BK (Bangladesh)	$125
ES (Ethiopia)	$130.5
IN (India)	$717
NK (Nigeria)	$500
NN (Nigeria)	$1100
NW (Nigeria)	$1100
PC (Pakistan)	$180
PP (Pakistan)	$500
RU (Rwanda)	$500
RK (Rwanda)	$200
ZAT (South Africa)	$1331

Supplementary Material Table 2 – The country level ethical approval for the BARNARDS study and sample/data collection. The site, committee name, principal investigator (PI), ethics reference and approval date are listed.

BARNARDS ethics approval documents and references.

Site	Committees	Named PI	Reference(s)	Approval date(s)
**BC**	Institute of Child Health	Samir Kumar Saha	BICH-ERC-4/3/2015	15/09/2015
**BK**	Institute of Child Health	Samir Kumar Saha	BICH-ERC-4/3/2015	15/09/2015
**ES**	Boston Children's Hospital	Grace Chan	IRB-P00023058	11/08/2016
**IN**	Institute of Cholera and Enteric Diseases and Institue of Post Graduate Medical Education and Research, IPGME&R Research Oversight Committee	Sulagna Basu	A-I/2016-IEC and Inst/IEC/2016/508	17/11/2016 and 04/11/2016
**NK**	Kano State Hospitals Management Board	Kenneth Iregbu	8/10/1437AH	13/07/2016
**NN**	Health Research Ethics Committee (HREC), National Hospital, Abuja	Kenneth Iregbu	NHA/EC/017/2015	27/04/2015
**NW**	Health Research Ethics Committee (HREC), National Hospital, Abuja	Kenneth Iregbu	NHA/EC/017/2015	27/04/2015
**PC**	University, Pakistan Institute of Medical Sciences (PIMS) Islamabad	Rabaab Zahra	NA, signed letter from Prof. Tabish Hazir	27/05/2015
**PP**	University, Pakistan Institute of Medical Sciences (PIMS) Islamabad	Rabaab Zahra	NA, signed letter from Prof. Tabish Hazir	27/05/2015
**RK**	Committee	Jean-Baptiste Mazarati	No342/RNEC/2015	10/11/2015
**RU**	Committee	Jean-Baptiste Mazarati	No342/RNEC/2015	10/11/2015
**ZAT**	Hospital, Research projects, Western Cape Government	Shaheen Mehtar	N15/07/063	04/12/2015 and 02/02/2016

Supplementary Material Table 3 – The primers used, PCR conditions and control strains for all PCRs performed within this study.

Primers, PCR conditions and control strains used in PCR experiments.

a) Primers, amplicons and control strains

Primers pair	Target	Sequence (5'-3')	Tan °C	Amplicon size (bp)	Reference	Control Strain
CTXM15- F<br>CTXM15- R	*bla* CTX-M-15	ATGCGCAAACGGCGGACGTA<br>CCCGTTGGCTGTCGCCCAAT	55	~600	Walsh group (Carvalho MJ)	*Escherichia coli* NCTC 13353
NDM-M-F<br>NDM-M-R	*bla* NDM	AGCTGAGCACCGCATT<br>CTCAGTGTCGGCATCAC	52-58	648	Walsh group (Hassan B)	Escherichia coli X (in-house control strain)
KPC-M-F<br>KPC-M-R	*bla* KPC	TAGTTCTGCTGTCTTGTCTC<br>CCGTCATGCCTGTTGTC	52-58	333	Walsh group (Hassan B)	*Klebsiella* sp. K10 (in-house control strain)
OXA-48-M-F<br>OXA-48-M-R	*bla* OXA-48 (and OXA-48-like genes: *bla* OXA-162, -163, -181, and possibly -204 & -232)	GGCGTAGTTGTGCTCTG<br>AAGACTTGGTGTTCATCCTT	52-58	155	Walsh group (Hassan B)	*K. pneumoniae* NCTC 13442

b) PCR conditions for *bla* CTX-M-15 survey

Number of cycles	Temperature	Time
1x	95°C	5 min
	94°C	30 s
30x	52°C	1 min
	72°C	1 min
1x	72°C	10 min

c) Multiplex-PCR conditions for *bla* NDM, *bla* KPC and *bla* OXA-48-like survey

Number of cycles	Temperature	Time
1x	95°C	5 min
	95°C	30 s
30x	61°C	30 s
	72°C	30 s
1x	72°C	10 min

Supplementary Material Table 4 – Information relating to the antibiotic susceptibility testing performed within this study.

a) List of antibiotics tested using the Kirby-Bauer disk diffusion method, following the EUCAST guidelines (v9, 2019)

Antibiotic	Acronym	Disk content (µg)
Amoxicillin	AML	10
Amoxicillin/clavulanate	AMX	20/10
Piperacillin/tazobactam	TZP	30/6
Cefotaxime	CTX	5
Cefatzidime	CAZ	10
Cefepime	FEP	30
Imipenem	IPM	10
Meropenem	MER	10
Ertapenem	ERT	10
Aztreonam	ATM	30
Gentamicin	GEN	10
Amikacin	AMK	30
Levofloxacin	LVX	5
Ciprofloxacin	CIP	5
Tigecycline	TGC	15
Trimethoprim/sulfamethoxazole	SxT	1.25/23.75
Nitrofurantoin	NIT	100
Fosfomycin	FOS	200 (50 glucose-6-phosphate)

Antibiotics disks from Liofilchem, Italy.

b) Quality control strains

Escherichia coli ATCC25922

Pseudomonas aeruginosa ATCC27853

Supplementary Material Table 4 (continued) – Information relating to the antibiotic susceptibility testing performed within this study.

Organisms	Amoxicillin	Amoxicillin/clavulanate	Piperacillin/tazobactam	Cefotaxime	Cefatzidime	Cefepime
Enterobacteriaceae	EUCAST V9; Enterobacterales	EUCAST V9; Enterobacterales	EUCAST V9; Enterobacterales	EUCAST V9; Enterobacterales	EUCAST V9; Enterobacterales	EUCAST V9; Enterobacterales
Pseudomonas spp.	EUCAST V9; Enterobacterales	EUCAST V9; Enterobacterales	EUCAST V9; *Pseudomonas* spp.	EUCAST V9; Enterobacterales	EUCAST V9; *Pseudomonas* spp.	EUCAST V9; *Pseudomonas* spp.
Acinetobacter spp.	EUCAST V9; Enterobacterales	EUCAST V9; Enterobacterales	EUCAST V9; *Pseudomonas* spp.	EUCAST V9; Enterobacterales	EUCAST V9; *Pseudomonas* spp.	EUCAST V9; *Pseudomonas* spp.
Stenotrophomonas maltophilia	EUCAST V9; Enterobacterales	EUCAST V9; Enterobacterales	EUCAST V9; *Pseudomonas* spp.	EUCAST V9; Enterobacterales	EUCAST V9; *Pseudomonas* spp.	EUCAST V9; *Pseudomonas* spp.
Aeromonas spp.	EUCAST V9; Enterobacterales	EUCAST V9; Enterobacterales	EUCAST V9; Enterobacterales	EUCAST V9; Enterobacterales	EUCAST V9; *Aeromonas* spp.	EUCAST V9; *Aeromonas* spp.
Sphingomonas sp.	EUCAST V9; Enterobacterales	EUCAST V9; Enterobacterales	EUCAST V9; *Pseudomonas* spp.	EUCAST V9; Enterobacterales	EUCAST V9; *Pseudomonas* spp.	EUCAST V9; *Pseudomonas* spp.
Shewanella sp.	EUCAST V9; Enterobacterales	EUCAST V9; Enterobacterales	EUCAST V9; *Pseudomonas* spp.	EUCAST V9; Enterobacterales	EUCAST V9; *Pseudomonas* spp.	EUCAST V9; *Pseudomonas* spp.
Bordetella spp.	EUCAST V9; Enterobacterales	EUCAST V9; Enterobacterales	EUCAST V9; *Pseudomonas* spp.	EUCAST V9; Enterobacterales	EUCAST V9; *Pseudomonas* spp.	EUCAST V9; *Pseudomonas* spp.
Brevundimonas spp.	EUCAST V9; Enterobacterales	EUCAST V9; Enterobacterales	EUCAST V9; *Pseudomonas* spp.	EUCAST V9; Enterobacterales	EUCAST V9; *Pseudomonas* spp.	EUCAST V9; *Pseudomonas* spp.
Alcaligenes spp.	EUCAST V9; Enterobacterales	EUCAST V9; Enterobacterales	EUCAST V9; *Pseudomonas* spp.	EUCAST V9; Enterobacterales	EUCAST V9; *Pseudomonas* spp.	EUCAST V9; *Pseudomonas* spp.

Organisms	Imipenem	Meropenem	Ertapenem	Aztreonam	Gentamicin	Amikacin
Enterobacteriaceae	EUCAST V9; Enterobacterales	EUCAST V9; Enterobacterales	EUCAST V9; Enterobacterales	EUCAST V9; Enterobacterales	EUCAST V9; Enterobacterales	EUCAST V9; Enterobacterales
Pseudomonas spp.	EUCAST V9; *Pseudomonas* spp.	EUCAST V9; *Pseudomonas* spp.	EUCAST V9; Enterobacterales	EUCAST V9; *Pseudomonas* spp.	EUCAST V9; *Pseudomonas* spp.	EUCAST V9; *Pseudomonas* spp.
Acinetobacter spp.	EUCAST V9; *Acinetobacter* spp.	EUCAST V9; *Acinetobacter* spp.	EUCAST V9; Enterobacterales	EUCAST V9; *Pseudomonas* spp.	EUCAST V9; *Acinetobacter* spp.	EUCAST V9; *Acinetobacter* spp.
Stenotrophomonas maltophilia	EUCAST V9; *Pseudomonas* spp.	EUCAST V9; *Pseudomonas* spp.	EUCAST V9; Enterobacterales	EUCAST V9; *Pseudomonas* spp.	EUCAST V9; *Pseudomonas* spp.	EUCAST V9; *Pseudomonas* spp.
Aeromonas spp.	EUCAST V9; Enterobacterales	EUCAST V9; Enterobacterales	EUCAST V9; Enterobacterales	EUCAST V9; *Aeromonas* spp.	EUCAST V9; Enterobacterales	EUCAST V9; Enterobacterales
Sphingomonas sp.	EUCAST V9; *Pseudomonas* spp.	EUCAST V9; *Pseudomonas* spp.	EUCAST V9; Enterobacterales	EUCAST V9; *Pseudomonas* spp.	EUCAST V9; *Pseudomonas* spp.	EUCAST V9; *Pseudomonas* spp.
Shewanella sp.	EUCAST V9; *Pseudomonas* spp.	EUCAST V9; *Pseudomonas* spp.	EUCAST V9; Enterobacterales	EUCAST V9; *Pseudomonas* spp.	EUCAST V9; *Pseudomonas* spp.	EUCAST V9; *Pseudomonas* spp.
Bordetella spp.	EUCAST V9; *Pseudomonas* spp.	EUCAST V9; *Pseudomonas* spp.	EUCAST V9; Enterobacterales	EUCAST V9; *Pseudomonas* spp.	EUCAST V9; *Pseudomonas* spp.	EUCAST V9; *Pseudomonas* spp.
Brevundimonas spp.	EUCAST V9; *Pseudomonas* spp.	EUCAST V9; *Pseudomonas* spp.	EUCAST V9; Enterobacterales	EUCAST V9; *Pseudomonas* spp.	EUCAST V9; *Pseudomonas* spp.	EUCAST V9; *Pseudomonas* spp.
Alcaligenes spp.	EUCAST V9; *Pseudomonas* spp.	EUCAST V9; *Pseudomonas* spp.	EUCAST V9; Enterobacterales	EUCAST V9; *Pseudomonas* spp.	EUCAST V9; *Pseudomonas* spp.	EUCAST V9; *Pseudomonas* spp.

Organisms	Levofloxacin	Ciprofloxacin	Tigecycline	Trimethoprim-sulfamethoxazole	Nitrofurantoin	Fosfomycin
Enterobacteriaceae	EUCAST V9; Enterobacterales	EUCAST V9; Enterobacterales	EUCAST V9; Enterobacterales - *E. coli*	EUCAST V9; Enterobacterales	EUCAST V9; Enterobacterales - *E.*	EUCAST V9; Enterobacterales
Pseudomonas spp.	EUCAST V9; *Pseudomonas* spp.	EUCAST V9; *Pseudomonas* spp.	EUCAST V9; Enterobacterales - *E. coli*	EUCAST V9; Enterobacterales	EUCAST V9; Enterobacterales - *E.*	EUCAST V9; Enterobacterales
Acinetobacter spp.	EUCAST V9; *Acinetobacter* spp.	EUCAST V9; *Acinetobacter* spp.	EUCAST V9; Enterobacterales - *E. coli*	EUCAST V9; *Acinetobacter* spp.	EUCAST V9; Enterobacterales - *E.*	EUCAST V9; Enterobacterales
Stenotrophomonas maltophilia	EUCAST V9; *Pseudomonas* spp.	EUCAST V9; *Pseudomonas* spp.	EUCAST V9; Enterobacterales - *E. coli*	EUCAST V9; *Stenotrophomonas ma*	EUCAST V9; Enterobacterales - *E.*	EUCAST V9; Enterobacterales
Aeromonas spp.	EUCAST V9; *Aeromonas* spp.	EUCAST V9; *Aeromonas* spp.	EUCAST V9; Enterobacterales - *E. coli*	EUCAST V9; *Aeromonas* spp.	EUCAST V9; Enterobacterales - *E.*	EUCAST V9; Enterobacterales
Sphingomonas sp.	EUCAST V9; *Pseudomonas* spp.	EUCAST V9; *Pseudomonas* spp.	EUCAST V9; Enterobacterales - *E. coli*	EUCAST V9; Enterobacterales	EUCAST V9; Enterobacterales - *E.*	EUCAST V9; Enterobacterales
Shewanella sp.	EUCAST V9; *Pseudomonas* spp.	EUCAST V9; *Pseudomonas* spp.	EUCAST V9; Enterobacterales - *E. coli*	EUCAST V9; Enterobacterales	EUCAST V9; Enterobacterales - *E.*	EUCAST V9; Enterobacterales
Bordetella spp.	EUCAST V9; *Pseudomonas* spp.	EUCAST V9; *Pseudomonas* spp.	EUCAST V9; Enterobacterales - *E. coli*	EUCAST V9; Enterobacterales	EUCAST V9; Enterobacterales - *E.*	EUCAST V9; Enterobacterales
Brevundimonas spp.	EUCAST V9; *Pseudomonas* spp.	EUCAST V9; *Pseudomonas* spp.	EUCAST V9; Enterobacterales - *E. coli*	EUCAST V9; Enterobacterales	EUCAST V9; Enterobacterales - *E.*	EUCAST V9; Enterobacterales
Alcaligenes spp.	EUCAST V9; *Pseudomonas* spp.	EUCAST V9; *Pseudomonas* spp.	EUCAST V9; Enterobacterales - *E. coli*	EUCAST V9; Enterobacterales	EUCAST V9; Enterobacterales - *E.*	EUCAST V9; Enterobacterales

Supplementary Material Table 5 – Long read sequencing analysis QC metrics for isolates selected as the reference genome for ST based SNP analysis

Isolate		BC-BR1421-3	BC-BR228-2
Species		*Escherichia coli*	*Escherichia coli*
ST		405	4684
Loci alleles		adk(35)	adk(6)
		fumC(37)	fumC(604)
		gyrB(29)	gyrB(3)
		icd(25)	icd(16)
		mdh(4)	mdh(11)
		purA(5)	purA(8)
		recA(73)	recA(6)
Long read metrics	Number of reads	39,449.00	72,491.00
	N50 long reads	15,375.00	18,155.00
Hybrid assembly metrics	# contigs	6	3
	Largest contig	5197174	4758536
	Total length	5556123	4942260
	GC (%)	50.61	50.8
	N50	5197174	4758536

Isolate		PP-BR254-2	PP-BR737-1	BC-MR44-1
Species		*Klebsiella pneu*	*Klebsiella pneum*	*Klebsiella pneumoniae*
ST		11	15	394
Loci alleles		gapA(3)	gapA(1)	gapA(2)
		infB(3)	infB(1)	infB(9)
		mdh(1)	mdh(1)	mdh(2)
		pgi(1)	pgi(1)	pgi(1)
		phoE(1)	phoE(1)	phoE(13)
		rpoB(1)	rpoB(1)	rpoB(1)
		tonB(4)	tonB(1)	tonB(82)
Long read metrics	Number of reads	37,492.00	81,332.00	26,187.00
	N50 long reads	13,491.00	9,450.00	13,726.00
Hybrid assembly metrics	# contigs	7	10	8
	Largest contig	5381779	5332228	5325299
	Total length	5828111	5914648	5738257
	GC (%)	56.98	56.7	56.89
	N50	5381779	5332228	5325299

Isolate		BC-BR406-2	BC-BR476-2	BC-MR78-3	BK-BR752-3
Species		*Enterobacter clc*	*Enterobacter hor*	*Enterobacter hormaecl*	*Enterobacter hormaechei*
ST		68	171	418	113
Loci alleles		dnaA(7)	dnaA(49)	dnaA(53)	dnaA(4)
		fusA(8)	fusA(21)	fusA(35)	fusA(22)
		gyrB(5)	gyrB(19)	gyrB(154)	gyrB(68)
		leuS(7)	leuS(44)	leuS(44)	leuS(69)
		pyrG(15)	pyrG(45)	pyrG(45)	pyrG(37)
		rplB(6)	rplB(12)	rplB(4)	rplB(4)
		rpoB(7)	rpoB(32)	rpoB(6)	rpoB(24)
Long read metrics	Number of reads	36,299.00	48,266.00	14,633.00	68,597.00
	N50 long reads	20,476.00	17,563.00	17,148.00	17,026.00
Hybrid assembly metrics	# contigs	4	4	5	10
	Largest contig	4775718	4616948	4696953	4516179
	Total length	4912294	5071364	4955331	4999801
	GC (%)	55.3	54.62	55.18	55.5
	N50	4775718	4616948	4696953	4516179

Supplementary Material Table 6 – Full list of BARNARDS consortium

Country	Forename	Surname	Position	Affiliation
Bangladesh	Samir	Saha	PI	Chittagong Medical College Hospital, Bangladesh
Bangladesh	Maksuda	Islam	scientist	Chittagong Medical College Hospital, Bangladesh
Bangladesh	Zabed	Bin-Ahmed	scientist	Chittagong Medical College Hospital, Bangladesh
Bangladesh	Wazir	Ahmed	clinician	Chittagong Medical College Hospital, Bangladesh
Bangladesh	Taslima	Begum	clinician	Chittagong Medical College Hospital, Bangladesh
Bangladesh	Mitu	Chowdhury	scientist	Chittagong Medical College Hospital, Bangladesh
Bangladesh	Shaila	Sharmin	scientist	Chittagong Medical College Hospital, Bangladesh
Bangladesh	Chumki	Rani Dey	Research nurse	Chittagong Medical College Hospital, Bangladesh
Bangladesh		Uttam	scientist	Chittagong Medical College Hospital, Bangladesh
Bangladesh	Abdul	Matin	clinician	Kumudini Women's Medical College, Bangladesh
Bangladesh	Sowmitra Ranjan	Chakraborty	scientist	Kumudini Women's Medical College, Bangladesh
Bangladesh	Sadia	Tasmin	clinician	Kumudini Women's Medical College, Bangladesh
Bangladesh	Dipa	Rema	Research nurse	Kumudini Women's Medical College, Bangladesh
Bangladesh	Rashida	Khatun	Research nurse	Kumudini Women's Medical College, Bangladesh
Bangladesh	Liza	Nath	Research nurse	Kumudini Women's Medical College, Bangladesh
Ethiopia	Nigatu	Balkachew	clinician	Department of Ob-Gyn, Saint Paul’s Hospital Millennium Medical College

Ethiopia	Delayehu	Bekele	Co-PI	Obstetrics and Gynaecology, Saint Paul's Hospital Millennium Medical College
Ethiopia	Katherine	Schaughency	Data manager	Saint Paul's Hospital Millennium Medical College
Ethiopia	Semaria	Solomon	laboratory	Department of Microbiology, Immunology and Parasitology, Saint Paul's Hospital Millennium Medical College
Ethiopia	Zenebe	Gebreyohanes	laboratory	Department of Microbiology, Immunology and Parasitology, Saint Paul's Hospital Millennium Medical College
Ethiopia	Rozina	Ambachew	laboratory	Department of Microbiology, Immunology and Parasitology, Saint Paul's Hospital Millennium Medical College
Ethiopia	Oludare	Odumade	clinician	Department of Pediatrics, Boston Children's Hospital Harvard Medical School
Ethiopia	Misgana	Haileselassie	nurse	Saint Paul's Hospital Millennium Medical College
Ethiopia	Grace	Chan	PI	Paediatrics, Harvard Medical School
Ethiopia	Abigail	Russo	Project manager	Global Health Research Program Manager, Boston Children's Hospital
Ethiopia	Redeat	Workneh	Project manager	Department of Pediatrics and Child Health, Saint Paul's Hospital Millennium Medical College
Ethiopia	Gesit	Metaferia	clinician	Department of Pediatrics, Saint Paul's Hospital Millennium Medical College
Ethiopia	Mahlet	Abayneh		Saint Paul's Hospital Millennium Medical College

Ethiopia	Yahya Zekaria	Mohammed		Birhan for Mothers and Children Program, Saint Paul's Hospital Millennium Medical College
Ethiopia	Tefera	Biteye		Birhan for Mothers and Children Program, Saint Paul's Hospital Millennium Medical College
Ethiopia	Alula	Teklu	scientist	Saint Paul's Hospital Millennium Medical College
Ethiopia	Wendimagegn	Gezahegn	clinician	Department of Pediatrics, Saint Paul's Hospital Millennium Medical College
India	Partha Sarathi	Chakravorty	Clinician, Co-Investigator	Professor and Head, Obstetrics & Gynecology, IPGMER & SSKM Hospital.
India	Suchandra	Mukherjee	Clinician, Co-Investigator	Professor and Head, Neonatology, IPGMER & SSKM Hospital.
India	Ranjan Kumar	Nandy	Co-PI	Scientist-F, Division of Bacteriology, ICMR-National Institute of Cholera and Enteric Diseases.
India	Samarpan	Roy	Data entry operator	ICMR-National Institute of Cholera and Enteric Diseases.
India	Anuradha	Sinha	SRF(Non-Medical)	Division of Bacteriology, ICMR-National Institute of Cholera and Enteric Diseases. Deputy manager(Vaccine), R&D, ZydusCadila.
India	Sharmi	Naha	SRF(Non-Medical)	Division of Bacteriology, ICMR-National Institute of Cholera and Enteric Diseases.
India	Sukla Saha	Malakar	Lower Division Clerk	ICMR-National Institute of Cholera and Enteric Diseases.

India	Siddhartha	Bose	Project Technician-III	ICMR-National Institute of Cholera and Enteric Diseases. Medical Laboratory Technologist, Swasthya Bhawan.
India	Monaki	Majhi	Project Technician-III	ICMR-National Institute of Cholera and Enteric Diseases. ICTC Lab Technician, Baruipur SD Hospital.
India	Subhasree	Sahoo	nurse	ICMR-National Institute of Cholera and Enteric Diseases.
India	Putul	Mukherjee	nurse	ICMR-National Institute of Cholera and Enteric Diseases.
India	Sumitra Kumari	Routa	nurse	ICMR-National Institute of Cholera and Enteric Diseases.
India	Chaitali	Nandi	nurse	ICMR-National Institute of Cholera and Enteric Diseases.
India	Sulagna	Basu	PI	Scientist F, Division of Bacteriology, ICMR-National Institute of Cholera and Enteric Diseases.
India	Bijan	Saha	Clinician, Co-Investigator	Associate Professor, Neonatology, IPGMER & SSKM Hospital.
India	Pinaki	Chattopadhyay	Clinician, Co-Investigator	Associate Professor, Neonatology, IPGMER & SSKM Hospital.
India	Sujoy	Roy	Multi-tasking Staff	ICMR-National Institute of Cholera and Enteric Diseases.
Nigeria	Fatima Zara Isa	Modibbo	clinician	54gene
Nigeria	Stella	Uwaezuoke	clinician	Federal Medical Centre Jabi, Abuja, Nigeria
Nigeria	Dilichukwu	Meduekwe	clinician	Wuse District Hospital, Nigeria
Nigeria	Khairiyya	Muhammad	laboratory	National Hospital Abuja, Nigeria

Nigeria	Queen	Nsude	laboratory	Wuse District Hospital, Nigeria
Nigeria	Ifeoma	Ukeh	laboratory	National Hospital Abuja, Nigeria
Nigeria	Mary-Joe	Okenu	laboratory	National Hospital Abuja, Nigeria
Nigeria	Akpulu	Chinenye	laboratory	54gene
Nigeria	Samuel	Yakubu	laboratory	National Hospital Abuja, Nigeria
Nigeria	Vivian	Asunugwo	laboratory	Wuse District Hospital, Nigeria
Nigeria	Folake	Aina	NYS Corp Member	National Hospital Abuja, Nigeria
Nigeria	Isibong	Issy	NYS Corp Member	National Hospital Abuja, Nigeria
Nigeria	Dolapo	Adekeye	NYS Corp Member	National Hospital Abuja, Nigeria
Nigeria	Adiele	Eunice	nurse	Wuse District Hospital, Nigeria
Nigeria	Abdulmlik	Amina	nurse	Wuse District Hospital, Nigeria
Nigeria	R	Oyewole	nurse	National Hospital Abuja, Nigeria
Nigeria	I	Oloton	nurse	National Hospital Abuja, Nigeria
Nigeria	BC	Nnaji	nurse	National Hospital Abuja, Nigeria
Nigeria	M	Umejiego	nurse	National Hospital Abuja, Nigeria
Nigeria	PN	Anoke	nurse	National Hospital Abuja, Nigeria
Nigeria	S	Adebayo	nurse	National Hospital Abuja, Nigeria
Nigeria	GO	Abegunrin	nurse	National Hospital Abuja, Nigeria
Nigeria	OB	Omotosho	nurse	National Hospital Abuja, Nigeria
Nigeria	R	Ibrahim	nurse	National Hospital Abuja, Nigeria

Nigeria	B	Igwe	nurse	National Hospital Abuja, Nigeria
Nigeria	M	Abroko	nurse	National Hospital Abuja, Nigeria
Nigeria	K	Balami	nurse	National Hospital Abuja, Nigeria
Nigeria	L	Bayem	nurse	National Hospital Abuja, Nigeria
Nigeria	C	Anyanwu	nurse	National Hospital Abuja, Nigeria
Nigeria	H	Haruna	nurse	National Hospital Abuja, Nigeria
Nigeria	J	Okike	nurse	National Hospital Abuja, Nigeria
Nigeria	K	Goroh	nurse	National Hospital Abuja, Nigeria
Nigeria	M	Boi-Sunday	nurse	National Hospital Abuja, Nigeria
Nigeria	Augusta	Ugafor	nurse	National Hospital Abuja, Nigeria
Nigeria	Maryam	Makama	nurse	National Hospital Abuja, Nigeria
Nigeria	Kaniba	Ndukwe	nurse	National Hospital Abuja, Nigeria
Nigeria	Anastesia	Odama	nurse	National Hospital Abuja, Nigeria
Nigeria	Hadiza	Yusuf	nurse	National Hospital Abuja, Nigeria
Nigeria	Patience	Wachukwu	nurse	National Hospital Abuja, Nigeria
Nigeria	Kachalla	Yahaya	nurse	National Hospital Abuja, Nigeria
Nigeria	Titus	Kalade Colsons	nurse	National Hospital Abuja, Nigeria
Nigeria	Mercy	Kura	nurse	National Hospital Abuja, Nigeria

Nigeria	Damilola	Orebiyi	nurse	National Hospital Abuja, Nigeria
Nigeria	Kenneth C.	Iregbu	PI	National Hospital Abuja, Nigeria
Nigeria	Chukwuemeka	Mmadueke	clinician	Wuse District Hospital, Nigeria
Nigeria	Lamidi	Audu	clinician	National Hospital Abuja, Nigeria
Nigeria	Nura	Idris	clinician	Director Kano State Health Trust Fund
Nigeria	Safiya	Gambo	clinician	Murtala Muhammad Specialist Hospital, Nigeria
Nigeria	Jamila	Ibrahim	clinician	Department of paediatric Muhammad Abdullahi Wase Teaching Hospital
Nigeria	Edwin	Precious	scientist	Department of Microbiology Aminu Kano Teaching Hospital
Nigeria	Ashiru	Hassan		Murtala Muhammad Specialist Hospital, Nigeria
Nigeria	Shamsudden	Gwadabe	scientist	Department of Physiology NorthWest University Kano
Nigeria	Adeola	Adeleye Falola	scientist	54gene
Nigeria	Muhammad	Aliyu	scientist	Murtala Muhammad Specialist Hospital, Nigeria
Nigeria	Amina	Ibrahim	scientist	Murtala Muhammad Specialist Hospital, Nigeria
Nigeria	Aisha Sani	Mukaddas	scientist	Murtala Muhammad Specialist Hospital, Nigeria
Nigeria	Rashida Yakubu	Khalid	scientist	Murtala Muhammad Specialist Hospital, Nigeria
Nigeria	Fatima Ibrahim	Alkali	scientist	Murtala Muhammad Specialist Hospital, Nigeria
Nigeria	Maryam Yahaya	Muhammad	scientist	Murtala Muhammad Specialist Hospital, Nigeria
Nigeria	Fatima Mohammad	Tukur	scientist	Murtala Muhammad Specialist Hospital, Nigeria

Nigeria	Surayya Mustapha	Muhammad	scientist	Department of Biotechnology Federal University Dutse
Nigeria	Adeola	Shittu	scientist	Murtala Muhammad Specialist Hospital, Nigeria
Nigeria	Murjanatu	Bello	scientist	Murtala Muhammad Specialist Hospital, Nigeria
Nigeria	Muhammad Abubakar	Hassan	scientist	Murtala Muhammad Specialist Hospital, Nigeria
Nigeria	Fatima Habib	Sa ad	scientist	Murtala Muhammad Specialist Hospital, Nigeria
Nigeria	Aishatu	Kassim	scientist	Murtala Muhammad Specialist Hospital, Nigeria
Pakistan	Haider	Shirazi	Co-PI	Neonatology Department, Pakistan Institute of Medical Sciences
Pakistan	Adil	Muhammad	laboratory	Department of Microbiology, Quaid-i-Azam University, Islamabad 45320, Pakistan
Pakistan	Rabaab	Zahra	PI	Department of Microbiology, Quaid-i-Azam University, Islamabad 45320, Pakistan
Pakistan	Syed Najeeb	Ullah	scientist	Department of Microbiology, Quaid-i-Azam University, Islamabad 45320, Pakistan
Pakistan	Muhammad	Hilal Jan	project manager	Department of Microbiology, Quaid-i-Azam University, Islamabad 45320, Pakistan
Pakistan	Rubina	Kamran	clinician	Pakistan Institute of Medical Sciences
Pakistan		Sajana	research nurse	Pakistan Institute of Medical Sciences
Pakistan	Jazba	Saeed	research nurse	Pakistan Institute of Medical Sciences
Pakistan	Noreen	Maqsood	research nurse	Pakistan Institute of Medical Sciences

Pakistan	Maria	Zafar	research nurse	Pakistan Institute of Medical Sciences
Pakistan	Saraeen	Sadiq	research nurse	Pakistan Institute of Medical Sciences
Pakistan	Sumble	Ahsan	research nurse	Pakistan Institute of Medical Sciences
Pakistan	Madiha	Tariq	scientist	Pakistan Institute of Medical Sciences
Pakistan	Sidra	Sajid	scientist	Pakistan Institute of Medical Sciences
Pakistan	Hasma	Mustafa	scientist	Pakistan Institute of Medical Sciences
Pakistan	Anees-ur	Rehman	local project manager	Pakistan Institute of Medical Sciences
Pakistan	Atif	Muhammad	local project manager	Pakistan Institute of Medical Sciences
Pakistan	Gahssan	Mehmood	local project manager	Pakistan Institute of Medical Sciences
Pakistan	Mahnoor	Nisar	local project manager	Pakistan Institute of Medical Sciences
Pakistan	Shermeen	Akif	local project manager	Pakistan Institute of Medical Sciences
Pakistan	Tahira	Yasmeen	local project manager	Pakistan Institute of Medical Sciences
Pakistan	Sabir	Nawaz	community coordinator	Pakistan Institute of Medical Sciences
Pakistan	Anam Shanal	Atta	community coordinator	Pakistan Institute of Medical Sciences
Pakistan	Mian	Laiq-ur-Rehman	community coordinator	Pakistan Institute of Medical Sciences
Pakistan	Robina	Kousar	community nurse	Pakistan Institute of Medical Sciences
Pakistan	Kalsoom	Bibi	community nurse	Pakistan Institute of Medical Sciences
Pakistan	Kosar	Waheed	community nurse	Pakistan Institute of Medical Sciences

Pakistan	Zainab	Majeed	community nurse	Pakistan Institute of Medical Sciences
Pakistan	Ayesha	Jalil	data support	Pakistan Institute of Medical Sciences
Rwanda	Espoir	Kajibwami	clinician	Kabgayi Hospital, Kabgayi, Gitarama, Rwanda
Rwanda	Aniceth	Rucogoza	laboratory	Microbiology Laboratory Director, National Reference Laboratory Rwanda.
Rwanda	Innocent	Nzabahimana	laboratory	The University Teaching Hostpial (CHUK), Kigali, Rwanda
Rwanda	Mazarati	Jean-Baptiste	PI	Head of Biomedical Services National Reference Laboratory, Kigali Rwanda
Rwanda	Lucie	Gaju	project manager	The University Teaching Hostpial (CHUK), Rwanda
Rwanda	Kankundiye	Riziki	nurse/scientis t	The University Teaching Hostpial (CHUK), Kigali, Rwanda
Rwanda	Brigette	Uwamahoro	nurse/scientis t	The University Teaching Hostpial (CHUK), Kigali, Rwanda
Rwanda	Rachel	Uwera	nurse/scientis t	The University Teaching Hostpial (CHUK), Kigali, Rwanda
Rwanda	Eugenie	Nyiratuza	nurse/scientis t	The University Teaching Hostpial (CHUK), Kigali, Rwanda
Rwanda	Kumwami	Muzungu	nurse/scientis t	The University Teaching Hostpial (CHUK), Kigali, Rwanda
Rwanda	Violette	Uwitonze	nurse/scientis t	Kabgayi Hospital, Kabgayi, Gitarama, Rwanda
Rwanda	Marie C	Horanimpundu	nurse/scientis t	Kabgayi Hospital, Kabgayi, Gitarama, Rwanda

Rwanda	Francine	Nzeyimana	nurse/scientist	Kabgayi Hospital, Kabgayi, Gitarama, Rwanda
Rwanda	Prince	Mitima	nurse/scientist	Kabgayi Hospital, Kabgayi, Gitarama, Rwanda
South Africa	Angela	Dramowski	clinician	Department of Paediatrics and child health, Stellenbosch University, Stellenbosch, Cape Town
South Africa	Andrew	Whitelaw	Co-PI	Division of Medical Microbiology, Stellenbosch University, Tygerberg Hospital, Cape Town
South Africa	Lauren	Paterson	laboratory	Division of Medical Microbiology, Stellenbosch University, Tygerberg Hospital, Cape Town
South Africa	Mary	Frans	nurse	Tygerberg Hospital, Cape Town
South Africa	Marvina	Johnson	nurse	Tygerberg Hospital, Cape Town
South Africa	Eveline	Swanepoel	nurse	Tygerberg Hospital, Cape Town
South Africa	Zoleka	Bojana	nurse	Tygerberg Hospital, Cape Town
South Africa	Mieme	du Preez	nurse	Tygerberg Hospital, Cape Town
South Africa	Shaheen	Mehtar	PI	Faculty of Medicine and Health Sciences, Unit for Infection Prevention and Control, Stellenbosch University
South Africa	Andre	Bulabula	scientist	Faculty of Medicine and Health Sciences, Unit for Infection Prevention and Control, Stellenbosch University

The Netherlands	Feiyan	Liu	scientist	Leiden Academic Centre for Drug Research, Leiden University, Leiden, The Netherlands
The Netherlands	Johan GC	van Hasselt	scientist	Leiden Academic Centre for Drug Research, Leiden University, Leiden, The Netherlands
UK	Timothy	Walsh	PI	Institute of Infection and Immunity, School of Medicine, Cardiff University
UK	Kirsty	Sands	scientist	Institute of Infection and Immunity, School of Medicine, Cardiff University
UK	Maria	Carvalho	scientist	Institute of Infection and Immunity, School of Medicine, Cardiff University
UK	Rebecca	Milton	project manager	Institute of Infection and Immunity, School of Medicine, Cardiff University
UK	Kathryn	Thomson	scientist	Institute of Infection and Immunity, School of Medicine, Cardiff University
UK	Edward	Portal	scientist	Institute of Infection and Immunity, School of Medicine, Cardiff University
UK	Jordan	Mathias	scientist	Institute of Infection and Immunity, School of Medicine, Cardiff University
UK	Calie	Dyer	Data manager	Institute of Infection and Immunity, School of Medicine, Cardiff University
UK	Ana	Ferreira	scientist	Institute of Infection and Immunity, School of Medicine, Cardiff University

UK	Robert	Andrews	scientist	Institute of Infection and Immunity, School of Medicine, Cardiff University
UK	John	Watkins	scientist	Institute of Infection and Immunity, School of Medicine, Cardiff University
UK	David	Gillespie	scientist	Centre for Trials Research, Cardiff University
UK	Kerry	Hood	Co-PI	Centre for Trials Research, Cardiff University
UK	Katie	Taiyai	scientist	Centre for Trials Research, Cardiff University
UK	Nigel	Kirby	scientist	Centre for Trials Research, Cardiff University
UK	Maria	Nieto	scientist	Institute of Infection and Immunity, School of Medicine, Cardiff University
UK	Thomas	Hender	scientist	Institute of Infection and Immunity, School of Medicine, Cardiff University
UK	Patrick	Hogan	scientist	Institute of Infection and Immunity, School of Medicine, Cardiff University
UK	Habiba	Saif	scientist	Institute of Infection and Immunity, School of Medicine, Cardiff University
UK	Brekhna	Hassan	scientist	Institute of Infection and Immunity, School of Medicine, Cardiff University
UK	Ellis	Jones	scientist	Institute of Infection and Immunity, School of Medicine, Cardiff University
UK	Matthew	Barrell	scientist	Institute of Infection and Immunity, School of Medicine, Cardiff University

UK	Ian	Boostrom	scientist	Institute of Infection and Immunity, School of Medicine, Cardiff University
UK	Francis	Frayne	scientist	Institute of Infection and Immunity, School of Medicine, Cardiff University
UK	Jessica	Rees	scientist	Institute of Infection and Immunity, School of Medicine, Cardiff University
UK	Lim	Jones	clinician	Specialist Antimicrobial Chemotherapy Unit, Public Heath Wales, Cardiff, UK
UK	Susanna	Dunachie	scientist	Centre for Tropical Medicine and Global Health, Nuffield Department of Medicine, University of Oxford
UK	Brad	Spiller	scientist	Institute of Infection and Immunity, School of Medicine, Cardiff University
UK	Julian	Parkhill	scientist	Cambridge Infectious Diseases, Department of Veterinary Medicine, Cambridge University
