# Supplementary material for: Antibiotic resistance genes in the gut microbiota of mothers and linked neonates with or without sepsis from low- and middle-income countries
Source: Nat Microbiol. 2022 Aug 4;7(9):1337–47. doi: 10.1038/s41564-022-01184-y (PMC9417982; doi:10.1038/s41564-022-01184-y)
Supplement: Supplementary file 2 — Reporting summary [file 41564_2022_1184_MOESM2_ESM.pdf]

nature research

Corresponding author(s): Maria J Carvalho

Last updated by author(s): Jun 17, 2022

Reporting Summary

Nature Research wishes to improve the reproducibility of the work that we publish. This form provides structure for consistency and transparency in reporting. For further information on Nature Research policies, see our Editorial Policies and the Editorial Policy Checklist.

Statistics

For all statistical analyses, confirm that the following items are present in the figure legend, table legend, main text, or Methods section.

n/a	Confirmed	
☐	☒	The exact sample size (*n*) for each experimental group/condition, given as a discrete number and unit of measurement
☐	☒	A statement on whether measurements were taken from distinct samples or whether the same sample was measured repeatedly
☐	☒	The statistical test(s) used AND whether they are one- or two-sided<br>*Only common tests should be described solely by name; describe more complex techniques in the Methods section.*
☐	☒	A description of all covariates tested
☐	☒	A description of any assumptions or corrections, such as tests of normality and adjustment for multiple comparisons
☐	☒	A full description of the statistical parameters including central tendency (e.g. means) or other basic estimates (e.g. regression coefficient) AND variation (e.g. standard deviation) or associated estimates of uncertainty (e.g. confidence intervals)
☐	☒	For null hypothesis testing, the test statistic (e.g. *F*, *t*, *r*) with confidence intervals, effect sizes, degrees of freedom and *P* value noted<br>*Give P values as exact values whenever suitable.*
☒	☐	For Bayesian analysis, information on the choice of priors and Markov chain Monte Carlo settings
☒	☐	For hierarchical and complex designs, identification of the appropriate level for tests and full reporting of outcomes
☒	☐	Estimates of effect sizes (e.g. Cohen's *d*, Pearson's *r*), indicating how they were calculated

Our web collection on statistics for biologists contains articles on many of the points above.

Software and code

Policy information about availability of computer code

Data collection	No software was used in the data collection. At the low middle income countries, research nurses completed questionnaires with the women approaching labor. These questionnaires were either transcribed onto paper, due to availability of resources/infrastructure, i.e. Internet access, and later uploaded into Bristol Online survey (BOS) or directly entered into BOS using a tablet device provided by the project.
Data analysis	CLIMB (v1.0)<br>Trimgalore (v0.4.3)<br>fastqc (v0.11.2)<br>MultiQC (v1.7)<br>Shovill and associated dependencies (v0.9.0)<br>quast (v.2.1)<br>Blast nt (https://blast.ncbi.nlm.nih.gov/Blast.cgi) (v2.2.25)<br>PathogenWatch (v.3.13.10; https://pathogen.watch)<br>ABRicate (v0.9.7) and associated pipelines: NCBI and PlasmidFinder<br>Enterobase<br>BIGSbd (v1.25.1)<br>PubMLST<br>Prokka (v1.12)<br>Roary (v3.12.0)<br>FastTree (v3.12.0)<br>iTOL (v4)<br>snippy (v4.6.0)<br>Gubbins (v2.3.4)<br>IQ-tree (v2.0)<br>Guppy (v5.0.11)

Filtlong (v0.2.0)
Unicycler (v0.4.9)
Nanoplot (v1.19.0)
Bandage (v0.8.1)
PLSBD
ChunLab's online ANI calculator
Stata (v16.1)
R studio using packages tidyr (v1.2.0), ggpubr (v0.4.3), gridExtra (v2.3), and egg (v0.4.3)

For manuscripts utilizing custom algorithms or software that are central to the research but not yet described in published literature, software must be made available to editors and reviewers. We strongly encourage code deposition in a community repository (e.g. GitHub). See the Nature Research guidelines for submitting code & software for further information.

Data

Policy information about availability of data

All manuscripts must include a data availability statement. This statement should provide the following information, where applicable:
- Accession codes, unique identifiers, or web links for publicly available datasets
- A list of figures that have associated raw data
- A description of any restrictions on data availability

Sequences reads have been submitted to the European Nucleotide Archive (ENA) under the project number PRJEB39293. Individual accession numbers and additional genomics data can be accessed in the Supplementary Material Methods and Source Data for Fig. 3-5. Hybrid assemblies (Illumina and ONT) have been submitted to NCBI under the BioProject number PRJNA767644.

Databases used within this study:
VFDB: http://www.mgc.ac.cn/VFs/download.htm
NCBI: https://github.com/tseemann/abricate/tree/master/db/ncbi
Resfinder: https://github.com/tseemann/abricate/tree/master/db/resfinder
Plasmidfinder: https://bitbucket.org/genomicepidemiology/plasmidfinder/src/master
mlst: https://github.com/tseemann/mlst/tree/master/db/pubmlst
PLSBD: https://ccb-microbe.cs.uni-saarland.de/plsdb/

Field-specific reporting

Please select the one below that is the best fit for your research. If you are not sure, read the appropriate sections before making your selection.

☒ Life sciences ☐ Behavioural & social sciences ☐ Ecological, evolutionary & environmental sciences

For a reference copy of the document with all sections, see nature.com/documents/nr-reporting-summary-flat.pdf

Life sciences study design

All studies must disclose on these points even when the disclosure is negative.

Sample size	The sampling method was purposive and a formal sample size calculation was not conducted.<br>Based on previous studies led by PI Professor Timothy Walsh (unpublished studies/awaiting publication), BARNARDS anticipated the enrollment level between 500-2000 neonates per clinical site for the duration of the study (depending on geographical location i.e. smaller rural site would have a smaller catchment area).
Data exclusions	The following exclusion criteria was pre-defined: the sepsis case infant/mother sampling pair was excluded in the case of a still born. Following this, data was retrospectively excluded based on the following criteria:<br>- Incomplete questionnaire; missing multiple data points in the epidemiological dataset<br>- Mother asked for infant withdrawal<br>- Error/substantial inconsistencies in the questionnaire - laboratory sampling match up
Replication	No replicas were used in this study, as one sample was taken per enrolled subject.
Randomization	All women approaching labour and their neonates and women who recently were in labour and whose neonates (≤60 days old) were clinically diagnosed with sepsis, were enrolled onto the study following consent. This was an observational study with no experimental and control groups, hence randomization was not relevant to the study.
Blinding	Blinding was not relevant for the study as this was an observational study with no randomization used. In each site, all samples were sequentially coded.

Reporting for specific materials, systems and methods

We require information from authors about some types of materials, experimental systems and methods used in many studies. Here, indicate whether each material, system or method listed is relevant to your study. If you are not sure if a list item applies to your research, read the appropriate section before selecting a response.

Materials & experimental systems

n/a	Involved in the study
☒	☐ Antibodies
☒	☐ Eukaryotic cell lines
☒	☐ Palaeontology and archaeology
☒	☐ Animals and other organisms
☐	☒ Human research participants
☒	☐ Clinical data
☒	☐ Dual use research of concern

Methods

n/a	Involved in the study
☒	☐ ChIP-seq
☒	☐ Flow cytometry
☒	☐ MRI-based neuroimaging

Human research participants

Policy information about studies involving human research participants

Population characteristics

BARNARDS was a multi-site international prospective observational study including two recruitment pathways:
i.) Birth-Cohort: All mothers in labour admitted to clinical-sites were recruited prospectively and their infant(s) followed up until 60-days old or death.
ii.) Infant Admissions (IA): Infant(s) admitted to clinical-sites showing signs of suspected sepsis in the first 60-days of life until 60- days old or death.

For this study, isolates recovered from mothers' and neonates' rectal samples were included irrespective of cohort pathway.
General population characteristics of the mothers' (outside of the scope of this manuscript): <10% previously had stillbirth, approx. 25% were first time mothers', 75% were aged between 21-35 years old.
Infants' presenting with sepsis were followed up for 60 days of life. Around 46% of neonates were 14 days old or less. Onset of sepsis was recorded, early onset (EOS) <72h, and late onset (LOS) >72h. Other population characteristics can be found in: Milton, R. et al. Neonatal sepsis and mortality in low-income and middle-income countries from a facility-based birth cohort: an international multisite prospective observational study. The Lancet Global Health 10, e661–e672 (2022).
Enrolled participants were not genotyped.

Recruitment

BARNARDS recruited from 12 clinical sites from Rwanda, Bangladesh, Ethiopia, Nigeria, Pakistan, India and South Africa. Where possible, large public hospitals were chosen. Recruitment took place between Nov 12, 2015, and Feb 1, 2018.
All women approaching labour and their neonates, and women who recently were in labour and whose neonates (≤60 days old) were clinically diagnosed with sepsis, were enrolled onto the study following consent. Consent was collected by trained research staff and using local languages. Neonates were then enrolled into the study.
Additionally, neonates not born within the clinical sites that were admitted with clinical signs of sepsis were also enrolled into the study following consent from the mother. The corresponding mothers were also enrolled into the study for the collection of samples and demographic data.
Neonatal follow-up was carried out at day 3, 7, 14, 28, and 60 by research nurses either face-to-face or by telephone.
Neonates remained in the study until 60 days old, withdrawal, or death.
This study incorporated two recruitment pathways to include both neonates born within the clinical sites, and also neonates in the larger catchment areas presenting to the hospital with signs of sepsis.
Women were approached and recruitment was totally dependent on their consent, so no selection bias was expected.

Ethics oversight

Site committees Named PI Reference(s) Approval date(s)
BC - Ethical Review Committee, Bangladesh Institute of Child Health Samir Kumar Saha BICH-ERC-4/3/2015 15/09/2015
BK - Ethical Review Committee, Bangladesh Institute of Child Health Samir Kumar Saha BICH-ERC-4/3/2015 15/09/2015
ES - Boston Children's Hospital Grace Chan IRB-P00023058 11/08/2016
IN - Institutional Ethics Committee, National Institute of Cholera and Enteric Diseases and Institue of Post Graduate Medical Education and Research, IPGME&R Research Oversight Committee Sulagna Basu A-I/2016-IEC and Inst/IEC/2016/508 17/11/2016 and 04/11/2016
NK - Kano State Hospitals Management Board Kenneth Iregbu 8/10/1437AH 13/07/2016
NN - Health Research Ethics Committee (HREC), National Hospital, Abuja Kenneth Iregbu NHA/EC/017/2015 27/04/2015 NW - Health Research Ethics Committee (HREC), National Hospital, Abuja Kenneth Iregbu NHA/EC/017/2015 27/04/2015 PC - Shaheed Zulfiqar Ali Bhutto Medical University, Pakistan Institute of Medical Sciences (PIMS) Islamabad Rabaab Zahra NA, signed letter from Prof. Tabish Hazir 27/05/2015
PP - Shaheed Zulfiqar Ali Bhutto Medical University, Pakistan Institute of Medical Sciences (PIMS) Islamabad Rabaab Zahra NA, signed letter from Prof. Tabish Hazir 27/05/2015
RK - Republic of Rwanda, National Ethics Committee Jean-Baptiste Mazarati No342/RNEC/2015 10/11/2015
RU - Republic of Rwanda, National Ethics Committee Jean-Baptiste Mazarati No342/RNEC/2015 10/11/2015
ZAT - Stellenbosch University and Tygerberg Hospital, Research projects, Western Cape Government Shaheen Mehtar N15/07/063 04/12/2015 and 02/02/2016

Ethical approval was obtained at each of the seven participating countries
Bangladesh: Ethical Review Committee, Bangladesh Institute of Child Health (BICH-ERC-4/3/2015), Ethiopia: Boston Children's Hospital (IRB-P00023058), India: Institutional Ethics Committee, National Institute of Cholera and Enteric Diseases and Institute of Post Graduate Medical Education and Research, IPGME&R Research Oversight Committee (A-I/2016-IEC and Inst/IEC/2016/508), Nigeria: Kano State Hospitals Management Board (8/10/1437AH), Health Research Ethics Committee (HREC) and National Hospital, Abuja (NHA/EC/017/2015), Pakistan: Shaheed Zulfiqar Ali Bhutto Medical University, Pakistan Institute of Medical Sciences (PIMS) Islamabad (Ref No NA, signed letter from Prof. Tabish Hazir). Rwanda: Republic of Rwanda, National Ethics Committee (No342/RNEC/2015), South Africa: Stellenbosch University and Tygerberg Hospital, Research

projects, Western Cape Government (N15/07/063). All approval dates are listed in the Supplementary Material Table 2. In local languages, research nurses provided mothers with study information and collected consent for mother and/or neonatal enrolment. Informed consent was obtained in writing unless this was not possible (due to literacy barriers), and oral consent was collected from the mothers by trained researchers. Oral consent was documented by the participant signing/marking the consent form.

Note that full information on the approval of the study protocol must also be provided in the manuscript.
